# Labor pain control and associated factors among women who gave birth at Leku primary hospital, southern Ethiopia

**DOI:** 10.1186/s13104-019-4645-x

**Published:** 2019-09-23

**Authors:** Melese Siyoum, Shewangizaw Mekonnen

**Affiliations:** 1 Department of Midwifery, College of Medicine and Health Sciences, Hawassa University, P.O. box 1560, Hawassa, Ethiopia; 2 School of Nursing, College of Medicine and Health Sciences, Hawassa University, P.O. box 1560, Hawassa, Ethiopia

**Keywords:** Labor analgesia, Labor pain, Labor pain control, Southern Ethiopia, Leku

## Abstract

**Objective:**

To assess labor pain control and associated factors among women who give birth at Leku primary hospital, southern Ethiopia, 2018/19. A systematic random sampling technique was used to select 404 mothers who gave birth at Leku hospital during the data collection period. Data were collected by two first degree midwives immediately after delivery using Labor Agentry Scale (LAS).

**Results:**

In this study, 404 mothers were participated making the response rate of 100%. Among the participants, 104 (25.7%) of mothers reported Mild control of labor pain. Maternal age of 19 to 24 year AOR = 5.85 (95% CI 2.14, 15.98), being farmer AOR = 2.5 (1.14, 5.57), primi-para AOR = 0.13 (0.06, 0.3), good family support AOR = 2.8 (1.49, 5.3), short duration of labor (< 12 h) AOR = 3.2 (1.65, 6.23) and history of pregnancy loss AOR = 0.06 (0.03, 0.14) were significantly associated with greater control of labor pain. In general, compared to other studies, the level of labor pain control is good in this study area. Enhancing factors of labor pain control have to be strengthened to increase greater control of labor pain. Qualitative research is highly recommended to identify cultural factors related to labor pain control and management.

## Introduction

Pain during labor and childbirth is a unique and the most severe pains event in women’s life. The extent to which a woman feels in control of pain during labor is an important indicator of maternal emotional wellbeing in childbirth [1]. More than 90% of the tension and stress during the pregnancy period is related to childbirth [2]. Loss of labor pain control was reported by 54.6% of women in the Netherland [3]. A study conducted in Sweden showed that 41% of participants reported labor pain as the worst experience that they have [4].

Labor pain is thought to have both physiological and psychological origin [5]. The physiological origin of labor pain is uterus contractions or cervical dilation [6] and psychological factors like stress, anxiety, and fear were shown to associated with labor pain [6, 7]. Pain stimulates the sympathetic nervous system, which causes an increase in the heart rate, blood pressure, sweat production, endocrine hyper-function, and delays the patient’s prognosis [8]. Poorly controlled labor pain resulted in negative or traumatic childbirth experiences [3].

Childbirth experiences/feeling control of labor pain/can be affected by internal and external factors of the women. Internal factors like attitude towards staff [9], attitude toward the experience of pain (perceiving), motivation towards childbearing and education about childbirth affects the extent of labor pain and feeling of control [10]. Those women who have learned how to experience safe childbirth showed low levels of stress as compared to their counterparts (control group) (p = 0.002) [11]. Labor pain tolerance is also affected by an individual’s endurance, acceptance of the pain and physical condition [12]. Only few works of the literatures indicated the factors for positive labor pain control. Therefore, this paper was aimed to assess women’s control of labor pain and associated factors among women who gave birth.

## Main text

### Study area and period

An institution-based cross-sectional study was conducted in Leku primary hospital, southern Ethiopia from December 2018 to March 30/2019. All women who present with singleton pregnancies at full-term (37–42 weeks) were included in the study while laboring women with known obstetric, medical or psychological problems were excluded. Systematic random sampling techniques were applied to select 404 study participants among post-partial mothers. In this study labor pain tolerance (self-control) was measured as “Greater control of pain” if women’s response score is more than a mean value of Labor Agentry Scale (LAS) and “Mild control of pain” if women’s answer score is less than a mean value of Labor Agentry Scale (LAS).

### Data collection techniques and tools

After the birth of the baby, each woman’s medical card was used to select participants who satisfy the eligibility criterion. Information about length (duration) of labor, type of labor (spontaneous/induced), mode of delivery, presence of complications, and details of the newborn were obtained from this medical registration. Postpartum women were interviewed in the postnatal unit within 24 h of vaginal delivery or 48 h after cesarean delivery for their feeling of labor pain using Labor Agentry Scale (LAS). Labour Agentry Scale (LAS) is a self-report scale designed to

measure feelings of control during childbirth which has a 10-item tool and each question (item) has a seven-point scale from one “almost always” to seven [7] “never”. The questions tend to measure the extent to which the mother felt she was in “tense, confident, important, lost self-control, fearful, relaxed, helpless, failure, in good behavior and have support from someone”. This tool was initially developed by Hodnett and Simmons Tropea. Factors associated with labor pain control were analyzed using binary and multivariable logistic regressions.

### Results

#### Socio-demographic characteristics

A total of 404 post-partial mothers were participated in this study making a response rate of 100%. The minimum and maximum ages of participants were 15 and 40 years respectively with a mean and standard deviation (± SD) of 24.46 ± 4.75 years. In this study majority of the participants were were married 385 (95.3%), Sidama ethnicity 336 (83.2%), protestant in religion 321 (79.5%), the majority of women were between 19 and 29 years of age (66.6%), only 35.9 of participants attended high school and above, 233 (57.7%) of them were from rural and 141 (34.9%) were farmers (see Table 1). Among the participants 381 (94.3%) has at least one Ante Natal Care visit, 196 (48.5%) were primigravida, 60 (14.9%) have a history of pregnancy loss (abortion or stillbirth), 208 (51.5%) were multi-gravida, 84 (20.8%) of pregnancies were unplanned, 122 (30.2%) have no/poor family support, 37 (9.2%) have history of depression, almost all 389 (96.3%) have no any complication, almost half of them 212 (52.5%) have no alive child, 23 of them gave birth by cesarean section and duration of labor was less than 12 h for 327 (80.9%) of participants (see Table 2).

**Table 1:** Socio-demographic characteristics of study participants on labor pain control at Leku general hospital, 2019

Variables	Category	Frequency (n = 404)	Percentage
Age	≤ 18	48	11.9
	19–24	130	32.2
	25–29	139	34.4
	≥ 30	87	21.5
Marital status	Single	19	4.7
	Married	385	95.3
Religion	Protestant	321	79.5
	Orthodox	54	13.4
	Muslim	18	4.5
	Catholic	09	2.2
	Adventist	2	0.5
Ethnicity	Sidama	336	83.2
	Amhara	36	8.9
	Oromo	10	2.5
	Silte	17	4.2
	Others	5	1.2
Residence	Urban	171	42.3
	Rural	233	57.7
Level of education	No formal education	31	7.7
	Primary (1–8)	228	56.4
	Secondary School (9–10)	85	21
	Above high school (> 10)	60	14.9
Occupation	House wife	87	21.5
	Employed (gov’t/NGO)	90	22.3
	Merchant	86	21.3
	Farmer	141	34.9

**Table 2:** Obstetrics characteristics of study participants on labor pain control at Leku general hospital, 2019

Variables	Category	Frequency (n = 404)	Percentage
Gravidity	Primigravida	196	48.5
	Multi-gravida	208	51.5
Frequency of ANC Visit	No ANC	23	5.71
	1–3 visit	246	60.89
	≥ 4 visit	135	33.4
Planned pregnancy	No	84	20.8
	Yes	320	79.2
Family support of the pregnancy	Poor	122	30.2
	Good	282	69.8
Pregnancy complication	No complication	389	96.3
	Yes	15	3.7
History of depression	No	367	90.8
	Yes	37	9.2
History of pregnancy loss	No	344	85.1
	Yes	60	14.9
Have alive child	No	212	52.5
	Yes	192	47.5
Mode of delivery	Vaginal	311	77
	Cesarean section	93	23
Duration of labor	≤ 12 h	327	80.9
	> 12 h	77	19.1

#### Level of labor pain control

Among the ten (10) questionnaires used to measure labor pain intensity, five of them were reversely recoded, to sum up, and calculate the mean value. Hence, the mean value of this study was 35. Accordingly, 104 (25.7%) of mothers reported that they have mild control of pain during labor and delivery (scored less than mean value to control labor). On Multivariate analysis, compared to age > 30 years only maternal age 19 to 24 year (AOR = 5.85; 95% CI 2.14, 15.98) associated with greater control of labor pain. Being farmer (AOR = 2.5; 95% CI 1.14, 5.57) compared to housewives, good family support (AOR = 2.8; 95% CI 1.49, 5.3), and short duration of labor (< 12 h) (AOR = 3.2; 95% CI 1.65, 6.23) were positively associated with greater control of labor pain. History of pregnancy loss (AOR = 0.06; 95% CI 0.03, 0.14) and primi-para (AOR = 0.13; 95% CI 0.06, 0.3) were negatively associated with greater control of labor pain (see Table 3).

**Table 3:** Factors associated with labor pain control among mothers who gave birth at Leku general hospital, SNNPR, Ethiopia, 2018/9

Variables	Level of pain Controls (n = 404)		COR (95% CI)	AOR (95% CI)	p-value
	Less control	Greater control			
Age of mother					
≤ 18	19	29	0.69 (0.34, 1.43)	2.9 (0.9, 9.7)	–
19–24	20	110	2.48 (1.28, 4.78)	5.85 (2.14, 15.98)*	0.001
25–29	38	101	1.2 (0.67, 2.15)	1.7 (0.8, 3.9)	–
≥ 30	27	60	1^r^	1^r^	
Marital status					
Single	9	10	1^r^	1^r^	–
Married	95	290	2.75 (1.08, 6.92)	1.1 (0.3, 4.04)	–
Occupation					
Farmer	24	117	2.3 (1.2, 4.3)	2.5 (1.14, 5.57)*	0.02
Merchant	22	64	1.4 (0.7, 2.7)	2.1 (0.9, 4.8)	
Gov’t employee	15	45	1.4 (0.7, 2.98)	1.9 (0.8, 4.9)	
Daily laborer	15	15	0.5 (0.2, 1.1)	0.8 (0.2, 2.2)	
House wife	28	59	1^r^	1^r^	
Gravida					
Primi gravida	61	135	0.58 (0.37, 0.9)	0.13 (0.06, 0.3)*	< 0.001
Multi gravida	43	165	1^r^	1^r^	
Family support					
Good	52	230	3.29 (2.1, 5.25)	2.8 (1.49, 5.3)*	< 0.001
Poor	52	70	1^r^	1^r^	
History of pregnancy loss					
No	61	283	1^r^	1^r^	
Yes	43	17	0.1 (0.05,0.16)	0.06 (0.03, 0.14)*	< 0.001
Duration of labor					
Less than 12 h	66	261	3.85 (2.3, 6.5)	3.2 (1.65, 6.23)*	0.001
Greater than 12 h	38	39	1^r^	1^r^	
Mode of delivery					
Vaginal	71	240	1.9 (1.13, 3.1)	1.34 (0.7, 2.6)	–
C/S	33	60	1^r^	1^r^	

* Significantly associated

### Discussion

In this study, 104 (25.7%) mothers reported that they failed to control labor pain during labor and delivery. This is not comparable with a finding from the Netherland and Swedish where 54.6% and 41% of post-partial mothers reported loss of labor pain control respectively [3, 4]. The difference might be due to a difference in culture and setting. Labor pain tolerance and expression of pain intensity is affected by culture, physical and psychological factors [9]. For instance, In Europe and America, women show a wide range of reactions against labor pain. However, in Korean culture, the women need to be quiet during delivery so they will not make their family ashamed [13].

The odds of greater control of pain among mothers who are 19 to 24 years old is almost six times higher than the odds mothers who are older than 30 years. This may be secondary to their physical endurance. Studies have identified that labor pain tolerance is affected by an individual’s endurance, acceptance of the pain and physical condition [12].

The odds of greater control of pain among primi-para mother is 87% less likely compared to multipara mothers. This is supported by previous studies where primi-paras complain about physical pain and discomfort more than multipara mothers [3, 14].

The odds of greater control of pain by women who have family support is almost three times higher than that of mothers who have no/poor family support. This is supported by a study conducted in Nepal where the presence of husband or other family members during childbirth is found to help mothers to cope with labor pain, [15–17].

The odds of greater control of pain is 2.5 times higher among farmers compared to the odds of pain control among housewives. This might be due to physical strength as farmers involve in hard work and frequent exercise than housewives.

Similar to study from the Netherland [3], the odds of greater control of pain among mothers who have a short duration of labor (≤ 12 h) were three times higher than their counterparts.

The odds of greater control of labor pain are 94% less likely for mothers who have a history of pregnancy loss compared to their counterparts. Higher intensity of labor pain is correlated with a history of abortion or stillbirth [3, 14] and having abnormal pregnancy increase labor pain [18].

## Limitations

The study was conducted only at one hospital, so that it may not representative the whole population.

## Data Availability

The dataset analyzed is available from the corresponding author on a reasonable request.

## Abbreviation

LAS: Labor Agentry Scale
